# Epigenetic Regulation in Prostate Cancer Progression

**DOI:** 10.1007/s40610-018-0095-9

**Published:** 2018-04-18

**Authors:** Katia Ruggero, Sonia Farran-Matas, Adrian Martinez-Tebar, Alvaro Aytes

**Affiliations:** 1 Programs of Molecular Mechanisms and Experimental Therapeutics in Oncology (ONCOBell), Catalan Institute of Oncology, Bellvitge Institute for Biomedical Research, Granvia de l’Hopitalet, 199 08908, L’Hospitalet de Llobregat, 08907 Barcelona, Spain; 2 Programs of Cancer Therapeutics Resistance (ProCURE), Catalan Institute of Oncology, Bellvitge Institute for Biomedical Research, L’Hospitalet de Llobregat, 08907 Barcelona, Spain

**Keywords:** Prostate cancer, Epigenetics, Transcriptional regulation, Chromatin biology, Androgen receptor, Drug targets

## Abstract

**Purpose of Review:**

An important number of newly identified molecular alterations in prostate cancer affect gene encoding master regulators of chromatin biology epigenetic regulation. This review will provide an updated view of the key epigenetic mechanisms underlying prostate cancer progression, therapy resistance, and potential actionable mechanisms and biomarkers.

**Recent Findings:**

Key players in chromatin biology and epigenetic master regulators has been recently described to be crucially altered in metastatic CRPC and tumors that progress to AR independency. As such, epigenetic dysregulation represents a driving mechanism in the reprograming of prostate cancer cells as they lose AR-imposed identity.

**Summary:**

Chromatin integrity and accessibility for transcriptional regulation are key features altered in cancer progression, and particularly relevant in nuclear hormone receptor-driven tumors like prostate cancer. Understanding how chromatin remodeling dictates prostate development and how its deregulation contributes to prostate cancer onset and progression may improve risk stratification and treatment selection for prostate cancer patients.

## Introduction

Prostate cancer has traditionally been seen as an aging-associated, low mutational load tumor with a tendency for genomic rearrangements and a particular dependency on the activity of the androgen receptor (AR). As such, treatment strategies have been focused on targeting the AR axis, either through inhibiting steroidogenic pathways and the production of testosterone, or by antagonizing the AR itself to prevent its nuclear translocation and the activation of its transcriptional network. While these strategies have doubtlessly improved survival for prostate cancer patients, they are not curative in many cases, and resistance eventually occurs in about 30% of patients, who develop castration-resistant prostate tumors (CRPC) for which limited treatment options exist. Moreover, under the CRPC definition, a pool of diverse disease presentations with variable outcomes exists, including neuroendocrine tumors.

Massive parallel sequencing of hundreds of tumor specimens from prostate cancer patients at different stages of cancer progression has provided an accurate picture of the landscape of genetic alterations that accompany cancer evolution in the prostate. Yet, despite several molecular classification systems for prostate tumors have been proposed, clear association with risk stratification remains to be provided. On the other hand, whether these genetic classifiers predict treatment outcome and to what extent genetic alterations in prostate cancer can be exploited for personalized therapies is yet to be proven. Interestingly, together with well-known drivers of cancer progression, an important number of new alterations have been described, with an intriguing enrichment of those affecting key players in chromatin biology and epigenetic master regulators (see a summary in Table 1). This is particularly relevant in metastatic CRPC and tumors that have transitioned to AR-independent phenotypes after progressing on the newest antiandrogen drugs.

**Table 1:** Summary of epigenetic master regulators implicated in prostate cancer

Gene name	Function in prostate cancer	References
Methyltransferases		
NSD2	H3K36 di-methyltransferase. Promotes prostate cancer tumorigenesis and progression. It is overexpressed in metastatic stage and associated with biochemical recurrence	[1••, 2, 3]
EZH2	H3K27 di- and tri-methyltransferase. Member of the polycomb repressive complex 2, crucial driver of prostate oncogenesis	[4, 5]
SUV39H1 (KMT1A) SETDB1 (KMT1E)	H3K9 tri methyltransferase. Enhance prostate cancer cell migration and invasion	[6, 7]
SUV39H2 (KMT1B)	H3K9 tri methyltransferase increases androgen-dependent transcriptional activity by interacting with the AR	[8]
SMYD3	H3K4 di- and methyltransferase, promotes cell proliferation and migration	[9, 10].
PRMT5	Drives prostate cancer cell growth through epigenetic inactivation of several tumor suppressors through histone arginine methylation at H4R3. Enhances AR-targeted gene expression	[11, 12•, 13]
Demethylases		
LSD1	H3K9 and H3K4 demethylase involved in prostate cancer recurrence, CRPC, and poor survival. Regulates AR transcriptional activity in a context-dependent manner	[14••, 15, 16•]
JARID1B (KDM5B)	H3K4 mono, di-, and tri-demethylase. AR coactivator regulating its transcriptional activity. Upregulated in prostate cancer tissues	[17, 18],
JARID1C (KDM5C)	H3K4 di- and tri-demethylase overexpressed in prostate cancer. Proposed as a predictive marker for therapy failure in patients after prostatectomy	[19].
JARID1D (KMD5D)	H3K4 di- and tri-demethylase. Suppress invasion and progression of prostate cancer. Low levels were associated with poor prognosis and resistance to docetaxel	[20, 21]
PHF8	H3K9, H3K27, and H4K20 demethylase. Transcriptional coactivator of AR. Promotes prostate cancer cell proliferation, migration, invasion, and neuroendocrine differentiation. Its expression highly correlated with poor prognosis and is induced by hypoxia	[22–29]
JMJD2A (KDM4A) JMJD2C (KDM4C)	H3K9 and H3K36 tri demethylases. Modulates AR transcriptional activity stimulating ligand-independent gene transcription via H3K9 demethylation	[30, 31]
JMJD1A (KDM3A)	H3K9 mono- and di-demethylase. Regulates AR activity by recruitment to target genes only in the presence of androgens	[32, 33]
JMJD2B (KDM4B),	H3K9 tri-demethylase, AR coactivator. Regulates AR transcriptional activity via demethylation activity and via inhibition of ubiquitination and increased AR stability	[34]
JMJD3 (KDM6B)	H3K27 di- and tri-demethylase overexpressed in metastatic prostate cancer	[35].
DNA methylation		
DNMTs	Control of transcriptional program during prostate cancer and CRPC progression	[36]
GSTP1	Silencing of GSP1 upon promoter hypermethylation is a potential prognostic biomarker and occurs early during prostate carcinogenesis	[37–39]
Histone acetylation		
P300	Histone acetyltransferase. Besides canonical histone acetylation activity, it acetylates the AR and enhances its transcriptional activity (coactivator) and drives prostate cancer growth	[40, 41]
SIRT1	Histone deacetylase; regulates cellular growth through AR deacetylation	[42, 43].
SIRT2	Histone deacetylase; its downregulation has been associated with increased acetylated H3K18 and poorer outcome and decreased sensitivity to androgen deprivation therapy	[44]
BET bromodomain epigenetic readers		
BRD4	Bromodomain and extra-terminal protein, interacts with AR and promote its activity and antiandrogen resistance	[45•, 46–48]
TRIM24	Epigenetic reader and transcription co-regulator, overexpressed in CRPC and associated to disease recurrence. Required for prostate cancer cell proliferation in CRPC	[49].
CHD1	H3K4me2-3 epigenetic reader whose loss is related with prostate cancer aggressiveness and DNA repair defects, thus sensitizing tumor cells to PARP inhibitors	[50, 51]
Pioneer transcription factors		
FOXA1	FOXA1 activity on chromatin results in increased accessibility and increased chromatin-bound AR. High FOXA1 expression leads to a restricted AR cistrome regulation. FOXA1 also has the potential to reprogram GATA2	[52, 53]
GATA2	GATA2 activity in human prostate cancer is strongly associated to AR levels and is hence considered a prostate cancer oncogene	[53–55]
Epigenetic regulators of lineage plasticity		
SOX2	Overexpressed TF in prostate cancer, regulating CRPC proliferation, and evasion of apoptosis. Promotes tumor metastasis by inducing EMT. Associated to NEPC emergence	[56–61, 62••, 63••]
MYC	Master regulator of prostate cancer transcriptional program. Associated with prostate cancer recurrence and poor prognosis	[64, 65]
MYCN	Driver of NEPC by inducing an EZH2-mediated transcriptional program	[64, 66]
Oncogenic pathways		
Hsp90	Initiates ERK signaling and leads to the recruitment of EZH2 to the E-cadherin promoter and repression of E-cadherin expression, driving EMT and invasion in prostate cancer cells	[67].
DAB2IP	Tumor suppressor Ras-GAP. Negatively controls Ras-dependent mitogenic signals and modulates TNFα/NF-κB, WNT/β-catenin, PI3K/AKT, and androgen receptors pathways	[68–70].
RB1	This tumor suppressor gene is commonly loss in metastatic and antiandrogen resistant prostate cancer and NEPC. Directly repress the expression of Sox2 and Ezh2	[71, 72, 63••]
ACK1	Tyrosine kinase correlated with poor prognosis and interacts with AR to drive ADT resistance and CRPC growth. Regulates transcription of AR and AR-v7 via epigenetic regulation	[72–74]

Here, we introduce key concepts to understand how epigenetic dysregulation is a plausible driving mechanism in the reprograming of prostate cancer cells as they lose AR-imposed identity. Beyond reviewing the current status of epigenetic biomarkers and classifiers and their clinical impact, we will discuss the scientific basis for therapeutic targeting master regulators of chromatin remodeling and integrity and the current state of epigenetic drugs for prostate cancer.

## DNA Methylation and Histone Modifications in Prostate Carcinogenesis

Perturbed DNA methylation patterns have long been reported during prostate cancer progression [75]. Among the most well-described alterations is the GSTP1 promoter hypermethylation and subsequent silencing [37], which is thought to occur early during prostate carcinogenesis [38] and has thus been proposed as a potential prognostic biomarker [39]. Yet, numerous other key genes have been implicated in DNA methylation changes. In fact, the promoter of the Androgen Receptor (AR) itself appears to be hypermethylated in up to 30% of CRPCs, resulting in the loss of AR expression [76]. Moreover, PTEN silencing is often a consequence of promoter CpG islands hypermethylation [77], while hypermethylation of the p16 tumor suppressor gene is associated with a proliferative advantage, thus contributing to carcinogenesis and disease progression [78]. Similarly, the hypomethylation and consequent upregulation of genes like heparanase and urokinase plasminogen activator (uPA) was reported to contribute to tumor cell invasion and metastasis [79]. More globally, DNA methylation signatures have been identified and proposed as molecular biomarkers of prostate cancer progression and treatment response [80].

Histone modifications also play an important role in the progression of many tumor types including prostate cancer. Lysine methyltransferases (KMT) and demethylases (KDM) are important epigenetic histone modifiers implicated in the control of gene transcriptional regulation as well as in non-histone protein posttranslational modifications and activity modulation [81]. More specifically, SUV39H1 (KMT1A) and SETDB1 (KMT1E) have been shown to enhance prostate cancer cell migration and invasion and to be upregulated in human prostate cancer specimens, and hence suggested as potential therapeutic targets [6], while SUV39H2 (KMT1B) interacts with the AR to increase androgen-dependent transcriptional activity [8]. Furthermore, levels of SETDB1 have been recently associated with prognosis and the development of bone metastases from prostate cancer [7]. Similarly, SET and MYND domain-containing protein 3 (SMYD3) has also been identified as an upregulated H3 and H4 lysine methyltransferase promoting cell proliferation and migration, thus emerging as a predictive marker of prostate cancer [10]. Alternatively, protein arginine methyltransferase 5 (PRMT5) was described as a prostate cancer oncogene driving cancer cell growth through epigenetic inactivation of several tumor suppressors [11] through histone arginine methylation at H4R3. PRMT5 has also recently been shown to enhance AR-targeted gene expression by arginine methylation and interaction with the transcription factor Sp1 [13].

Demethylases also play an important role in prostate cancer development. Lysine-specific demethylase 1 (LSD1/KDM1A) has been proposed as an oncogene whose overexpression has been positively correlated with the malignancy of many cancer types, including prostate [14••, 82], promoting carcinogenesis by multiple mechanisms. Increased LSD1 expression is associated with prostate cancer recurrence and poor survival and appears to have distinct functions in androgen-dependent [14••, 83] and refractory prostate cancer [15]. Recently, it was discovered that LSD1 is a co-regulator of vitamin D receptor activity in prostate cancer and its expression is correlated with shorter progression-free survival in primary and metastatic patients [84]. In a recent study, it was found that LSD1-mediated epigenetic reprogramming drives CRPC and was associated with the activation of CENPE, which was regulated by the co-binding of LSD1 and AR to its promoter region, which was associated with loss of RB1 [16•].

The overexpression of other histone demethylases (HDMs) has also been observed in prostate cancer. An exhaustive functional screen [27] identified 32 enzymes belonging to the family of JmjC domain-containing histone demethylases as critical for prostate cancer proliferation and survival. KDM5 family members are H3K4 demethylases; JARID1B (KDM5B) is upregulated in prostate cancer tissues and acts as an AR coactivator [17], while JARID1C (KDM5C), overexpressed in prostate cancer, emerged as a predictive marker for therapy failure in patients after prostatectomy [19]. JARID1D (KMD5D) was found to suppress the invasion and progression of prostate cancer cells; thus, it is highly downregulated in metastatic prostate tumors and those low levels were associated with poor prognosis [20]. In addition, KDM5 loss has been associated with resistance to docetaxel in prostate cancer [21]. The PHD-finger protein 8 (PHF8) is a histone demethylase and a transcriptional coactivator of AR via H4K20 demethylation [28]. Its expression, highly correlated with poor prognosis, is induced by hypoxia and promotes prostate cancer cell proliferation, migration and invasion [28], and neuroendocrine differentiation [29].

### The Histone Methyltransferase NSD2

NSD2 (nuclear receptor binding SET domain protein 2), also known as WHSC1 (Wolf-Hirschhorn syndrome candidate 1) and MMSET (multiple myeloma SET domain), is a member of the histone methyltransferase NSD family of proteins also including NSD1 and NSD3. NSD2 catalyzes the dimethylation of histone H3 at lysine 36 (H3K36me2), a permissive mark associated with open chromation conformation and active gene transcription [85]. NSD2 was first linked to oncogenesis by the involvement in the t(4; 14) translocation identified in up to 20% of multiple myeloma patients [86]. In the past years, NSD2 has been shown to be overexpressed in a variety of solid tumors including prostate cancer, where it has been found overexpressed in metastatic PCa compared to primary tumors and is associated with biochemical recurrence [1••]. Further In vitro studies strengthened the role of NSD2 in prostate cancer tumorigenesis; it has been shown that NSD2 modulates Twist family bHLH transcription factor 1 (TWIST1) to promote epithelial to mesenchymal transition and invasiveness in prostate cancer cell lines [2]. Moreover, Asangani and colleagues had reported that EZH2 mediates the overexpression of NSD2 and that the oncogenic properties of EZH2 are NSD2 dependent [3]. Interestingly, transcriptional targets of NSD2 in prostate cancer cells are highly enriched for components of the NF-kB-network, including IL-6, IL-8, survivin/Birc5, and VEGFA. In fact, NSD2 has been linked to constitutive activation of NF-kB signaling in CRPC, promoting cancer cell proliferation and survival via an autocrine positive loop in which NSD2 expression is in turn stimulated by inflammatory cytokines, such as TNFα and IL-6, via NF-kB [87].

Very recently, work from Li and collaborators showed that NSD2 is activated in PTEN null tumors by the AKT pathway and that its expression is required for metastatic progression. Mechanistically, AKT-mediated phosphorylation of NSD2 prevents its degradation by CRL4^Cdt2^ E3 ligase leading to NSD2 stabilization and overexpression. By directly inducing RICTOR expression, NSD2 mediates a positive feedback loop sustaining AKT signaling [1••].

Finally, NSD2 has been shown to physically interact with the AR DNA-binding domain and to be recruited to the enhancer region of the PSA gene and enhance AR transcriptional activity [88], suggesting that NSD2 might be implicated in resistant to ADT or androgen signaling inhibition. Of note is the recent identification of NSD2 as a candidate gene promoting androgen independence through an unbiased insertional mutagenesis screen [89]. In fact, unpublished data and data from our laboratory currently under peer-review for publication strongly suggest that NSD2 is an actionable mechanism in CRPC.

## Epigenetic Control of Androgen Receptor Activity

Histone modifying enzymes, and LSD1 in particular, are among the best-known modulators of AR transcriptional activity. LSD1 is an important enzyme involved in AR regulation and prostate cancer that interacts with AR and can stimulate [14••] or suppress [15] the transcriptional expression depending on promoter/enhancer context. This interaction promotes ligand-dependent transcription of AR target genes, resulting in enhanced tumor cell growth. Its coactivator activity seems to be associated with H3K9me1,2 demethylation leading to transcriptional de-repression of AR target genes [14••]. Intriguingly, LSD1 also plays a role as co-repressor, via H3K4me1,2 demethylation [90] and the recruitment of co-repressor complexes. This highlights the dual role of many chromatin remodelers and may explain why translating them to new therapeutics has so far been limited. A possible way forward may be to define the context specificities for this duality. For example, it has been shown that in high androgen levels, AR recruits LSD1 to mediate AR gene silencing [15]; however, this negative feedback loop is apparently disrupted in CRPC, where low androgen levels promote AR overexpression. Additionally, post-transcriptional modifications can regulate LSD1 activity and may become better targets; LSD1 phosphorylation [91] results in a switch of substrate from H3K4me1,2 to H3K9me1,2, and the promotion of its coactivator activity. Jumonji C domain-containing trimethyl lysine demethylases JMJD2A (KDM4A) and JMJD2C (KDM4C) also play a significant role in modulating AR transcriptional activity [30, 31], stimulating ligand-independent gene transcription via H3K9 demethylation. On the contrary, JMJD1A (KDM3A) recruitment to target genes only occurs in the presence of androgens, regulating AR activity and identifying KDM3A-dependent genes involved in androgen response, hypoxia, glycolysis, and lipid metabolism [33], again evidencing the complex balance between chromatin modifying enzymes in controlling different but interconnected cellular processes. Of note is the case of JMJD2B (KDM4B), which is an AR coactivator, emerging as a suitable therapeutic target for the treatment of prostate cancer. JMJD2B controls AR transcriptional activity via demethylation and inhibition of ubiquitination and increased AR stability [34]. Finally, JMJD3 (KDM6B) is progressively overexpressed in metastatic prostate cancer [35].

### Histone Acetylation and AR

Acetylated chromatin is generally associated to active transcription and the enzymes regulating this process are histone acetyltransferases (HAT) and deacetylases (HDAC). Accordingly, acetylated histone H3 in the vicinity of AR-bound chromatin has been shown to reduce androgen dependence in castration resistance models [92, 93]. That is the case for canonical HAT like p300 and CREB-binding protein, which, besides canonical histone acetylation activities, have been shown to acetylate the AR and enhance its transcriptional activity [40]. Importantly, two groups have recently independently developed small molecule inhibitors targeting p300/CBP. Lasko and colleagues reported a selective catalytic p300/CBP inhibitor able to downregulate the AR transcriptional program both in castration-sensitive and castration-resistant prostate tumors and to inhibit tumor growth in CRPC xenograft models [94], while Jin and colleagues found that targeting the p300/CBP bromodomain had remarkably similar effetcs [41]. More broadly, a recent study highlights the important role of histone acetylation in prostate cancer beyond active promoters via activation of AR associated enhancers and the increase in chromatin accessibility [95•].

Conversely, a variety of HDACs are also capable of deacetylating the AR and inhibit its activity, for example via regulation of heat-shock protein 90 (Hsp90), a chaperone controlling AR nuclear localization and activation through its acetylation/deacetylation, or sirtuin 1 (SIRT1), which regulates cellular growth through AR deacetylation [42, 43]. In fact, acetylation of H3K18, putatively via downregulation of SIRT2 deacetylase, has been associated to poorer outcome and decreased sensitivity to androgen deprivation therapy (ADT). Finally, at the mechanistic level, the Wu lab has recently demonstrated that HDAC inhibitors can suppress HMGA-driven EMT, reduce tumor growth and metastasis and, importantly, resensitize prostate cancer cells to [96].

## The Role of EZH2/Polycomb Repressive Complex in Prostate Cancer

The enhancer of zeste homolog 2 (EZH2) is a critical member of the Polycomb Repressive Complex 2 (PRC2) that regulates histone methylation mainly via lysine 27 at histone H3 (H3K27), a modification associated to transcriptional silencing [97] that is found upregulated in many tumor types. In prostate cancer, its elevated expression associates with poorer outcomes and has therefore been proposed as an oncogene [4, 98]. A major function of EZH2 is to repress lineage-specifying factors, thereby promoting stemness features [99], epithelial-mesenchymal transition (EMT), and ultimately metastatic progression [100]. A wealth of recent evidence has confirmed these previous observation in the prostate cancer field. Back-to-back recent articles in *Science* by the Sawyers and Goodrich groups demonstrated that lineage plasticity and neuroendocrine differentiation in androgen independence is partly driven by Ezh2 and Sox2 in prostate cancer mouse models carrying loss of function alleles for p53 and Rb tumor suppressors [62••, 63••]. This came to confirm two previous reports by Dardenne and colleagues [64] and by Xu and colleagues [101] showing that N-myc induces EZH2-driven neuroendocrine prostate cancer [64] and it cooperates with E2F1 in castration resistance [101].

Yet, EZH2 has also PRC2-independent roles as coactivator of transcription factors, including an AKT-dependent methylation of the AR, via PI3K/AKT phosphorylation of EZH2 at serine 21 [102], and modulation of AR recruitment to its target sites [103••]. Not surprisingly, EZH2 inhibitors are the focus of intensive development and have been widely tested in vivo [5] and in clinical trials (see Table 2 for details). Beyond a promising drug target, EZH2 and TOP2A have been proposed as prognostic as well as predictive biomarkers of treatment response against EZH2 inhibitors [104].

**Table 2:** Clinical trials for epigenetic drugs including prostate cancer patients

Trial ID	Drug	Phase	Conditions	Patients	Status
BET bromodomain inhibitors					
NCT02259114	OTX015/MK-8628	I	NUT midline carcinoma, triple negative breast cancer, non-small cell lung cancer (rearranged ALK or mut KRAS), CPRC, pancreatic ductal adenocarcinoma	47	Completed
NCT02698176		I	NUT midline carcinoma, triple negative breast cancer, non-small cell lung cancer, CRPC	13	Terminated
NCT01987362		I	Solid Tumors	120	Active
NCT02711956	ZEN003694	I	Metastatic CRPC (+enzalutamide)	58	Recruiting
NCT02705469		I	Metastatic CRPC	44	Active
NCT03266159	GSK525762	II	Solid tumors	150	Not recruiting
NCT02419417	BMS-986158	I/II	Advanced solid tumors	150	Recruiting
NCT02391480	ABBV-075	I	Advanced cancer, breast cancer, non-small, ell lung cancer, acute myeloid leukemia, multiple myeloma, prostate cancer, small-cell lung cancer, non-Hodgkins lymphoma	150	Recruiting
NCT02630251	GSK2820151	I	Advanced or recurrent solid tumors	60	Recruiting
NCT02369029	BAY 1238097	I	Neoplasms	8	Terminated
NCT02431260	INCB054329	I/II	Advanced cancer	69	Active, not recruiting
NCT02711137	INCB057643	I/II	Advanced cancer	230	Recruiting
NCT02607228	GS-5829	I/II	Metastatic CRPC (+enzalutamide)	132	Recruiting
NCT02711137	INCB057643	I/II	Advanced solid tumors and hematologic malignancy (+abiraterone)	420	Recruiting
EZH2 and PRC1/2 inhibitors					
NCT03213665	Tazemetostat	II	Advanced solid tumors, non-Hodgkin lymphoma, or histiocytic (EZH2, SMARCB1, or SMARCA4 mutations)	49	Recruiting
NCT01897571		I/II	Advanced solid tumors	420	Recruiting
NCT02875548		II	Advanced solid tumors	300	Recruiting
NCT03217253		I	Metastatic malignant solid neoplasm	48	Not recruiting
PRMT5 inhibitor					
NCT02900651	MAK683	I/II	Diffuse large B cell lymphoma, advanced solid tumors	113	Recruiting
LSD1/KDM1A inhibitors					
NCT02712905	INCB059872	I/II	Advanced cancer	180	Recruiting
DNMT inhibitors					
NCT01118741	Disulfiram		Prostate cancer	19	Completed
NCT00503984	Azacitidine	I/II	Metastatic CRPC (+docetaxel, prednisone)	22	Terminated
NCT00384839		II	CRPC	53	Completed
NCT02998567	Guadecitabine	I	Non-small cell lung cancer, CRPC (+pembrolizumab)	35	Not yet recruiting
HDAC inhibitors					
NCT01075308	Pracinostat (SB939)	II	Metastatic CRPC	32	Completed
NCT00670553		I	Prostate cancer, head and neck cancer, esophageal cancer	7	Completed
NCT00878436	Panobinostat (LBH589)	I/II	CRPC (+bicalutamide)	52	Completed
NCT00667862		II	Metastatic CRPC	35	Completed
NCT00663832		I	CRPC (+docetaxel and prednisone)	44	Completed
NCT00493766		I	CRPC (+docetaxel and prednisone)	16	Terminated
NCT00419536		I	CRPC (+docetaxel and prednisone)	108	Terminated
NCT00330161	Vorinostat (SAHA, MK0683)	II	Metastatic CRPC	29	Completed
NCT01174199		I	Metastatic CRPC	13	Terminated
NCT00589472		II	Primary prostate cancer (+bicalutamide.)	19	Completed
NCT00565227		I	Non-small-cell lung carcinoma, prostate cancer, bladder cancer, urothelial carcinoma	12	Terminated
NCT00511576	Mocetinostat (MGCD0103)	I	Breast cancer, lung cancer, prostate cancer, gastric cancer (+docetaxel)	54	Terminated
NCT00020579	Entinostat (MS-275)	I	Advanced solid tumors, lymphoma	75	Completed
NCT00413075	Belinostat (PXD101)	I	Advanced solid tumors, lymphoma	121	Completed
NCT00413322		I	Advanced solid tumors (+5-fluorouracil)	35	Completed

## Bromodomain-Containing Proteins in Prostate Cancer

Bromodomain-containing proteins are chromatin readers that recognized mono-acetylated histones and trigger chromatin remodeling to initiate transcription. Mutations and deregulation of BRD-containing proteins is a common feature of a variety of cancers. More than 50% of primary and metastatic prostate tumors and more than 70% of neuroendocrine prostate cancer present genomic alterations in any of the 42 known BRD-containing proteins [105]. Further, BRD-containing proteins have a diversity of catalytic and scaffolding functions and may act as transcription factors, transcriptional co-factors recruiting other proteins in the transcriptional complex, methyltransferases, HATs, Helicases, and ATP-dependent chromatin remodelers, therefore playing a central role in gene expression regulation [106].

The subgroup of BET proteins (bromodomain and extra-terminal), and in particular BRD4, have been the best characterized in prostate cancer, and several inhibitors of BET bromodomains have been developed and are currently in clinical trial (see Table 2). The conserved BET family includes BRD4, BRD2, BRD3, expressed ubiquitously, and BRDT, specifically expressed in the testis. BRD4 recognizes acetylated lysines at enhancers/superenhancer [107••, 108••] and recruits the elongation factor P-TEFb and stimulates RNA polymerase II-dependent transcription [109]. A provocative new finding by Zuber and colleagues with implications in risk assessment shows that tissue-specific SNPs in super-enhancer sequence bound by BRD4 are significantly associated with increased prostate cancer risk and show better enrichment for risk loci than AR [110].

BRD4 physically interacts with high-affinity with the N-terminal domain of AR leading to AR translocation into the nucleus and AR recruitment to target loci, promoting AR activity and expression of AR target genes in CRPC [45•]. A recent study showed that the small molecule BET inhibitor ABBV-075 could disrupt the recruitment of BRD4 at enhancer of AR target genes and repress their expression, whithout affecting AR protein levels [111]. Moreover, BET proteins have a role in resistance to antiandrogens and BET inhibitors can effectively resensitize resistant tumors to enzalutamide [112]. One of these mechanisms of resistance to antiandrogens is the upregulation of the glucocorticoid receptor (GR), and the co-option of the AR regulon, thus favoring CRPC progression by overcoming AR dependency [46, 47, 113].

Beyond AR signaling, BRD4 has been shown to bind to the truncated ERG (ERGΔ39) encoded by the TMPRSS2-ERG fusion, co-regulating the expression of ERG target genes in CRPC, thereby stimulating cell growth and invasion [114]. Additionally, SPOP, an E3 ligase substrate binding protein frequently mutated in prostate cancer, was also reported to target BET proteins for ubiquitination-mediated degradation. Interestingly, SPOP mutants fail to ubiquitinate BET proteins, leading to their stabilization and to resistance to BET inhibitors [48, 115]. This mechanism of resistance causes activation of AKT-mTORC1 signaling and consequently resistance to BET inhibitors can be overcome by combination with AKT inhibitors [116].

It is well known that one of the major aging-associated drivers of prostate carcinogenesis is oxidative stress and its impact on DNA [117]. Interestingly, Hussong and colleagues have recently established a link between BRD4 and oxidative stress response genes in prostate cancer, such as the KEAP1/NRF2 axis and HMOX1, and reactive oxygen species (ROS) production [118].

Other than BET, several BRD-containing proteins have been associated to prostate cancer progression and are at different validation stages for therapeutic targets in mCRPC. TRIM24, tripartite motif-containing protein 24, is an epigenetic reader and transcription co-regulator overexpressed in CRPC and associated to disease recurrence. Recurrent SPOP mutants stabilize TRIM24 [119], enhancing AR signaling and promoting tumor growth via binding with the proteins TIP60 and BRD7 [120], which has led to the proposition of TRIM24 as an essential gene for prostate cancer cell proliferation in CRPC [49].

Finally, the role of chromodomain proteins, and in particular chromodomain helicase DNA-binding protein 1 (CHD1), has in the recent years been elucidated in the context of prostate cancer progression. This H3K4me2-3 epigenetic reader has been reported mutated in 43% of Gleason 7 or higher prostate cancer tumors, associated with ETS gene fusion negative status [121] and its loss related with prostate cancer aggressiveness [50] and DNA repair defects, hence sensitizing tumor cells to PARP inhibitors [51]. More recently, Zhao and colleagues at the DePinho laboratory have demonstrated in PTEN null prostate tumors that CHD1 depletion dramatically suppressed cell proliferation, survival, and tumorigenic potential by activating the pro-tumorigenic TNF-NF-κB gene network [122].

## Pioneer Factors in Prostate Cancer Progression

Different from other DNA bound proteins and transcription factors, pioneer factors can access their targets in nucleosomes and in highly compacted chromatin regions, facilitating chromatin accessibility and the recruitment of additional TFs and co-TF and the transcriptional machinery [123]. Among paradigmatic pioneering factors are some of the members of the GATA and FoxA gene families, known mainly for their key role as chromatin-factors during early development [124–127].

The best-known pioneering factor for its role in prostate cancer is FOXA1. Through the interaction and recruitment of AR to chromatin site, FoxA1 defines and controls the AR cistrome resulting in context-dependent positive or negative regulation [52, 55, 128, 129]. In particular, because FOXA1 activity on chromatin results in increased accessibility [52] and increased chromatin-bound AR, high FOXA1 expression leads to a restricted AR cistrome regulation [53].

GATA genes, and GATA2 in particular, have proved to be crucial for prostate development via modulating AR function [54, 55]. However, despite the role is comparable to that of FoxA1, the mechanisms have shown to be quite different. GATA2 depletion did not seem to have a reprogramming effect on AR binding sites and in fact correlated with a downregulation in AR expression. Accordingly, GATA2 activity in human prostate cancer is strongly associated to AR levels and is hence considered a prostate cancer oncogene. Provocatively, it was found that FOXA1 also has the potential to reprogram GATA2 and act as a pioneering effect for both AR and GATA2, suggesting that FOXA1 regulates a transcriptional network that controls AR-mediated gene expression in prostate cancer [53].

## Lineage Plasticity in Prostate Cancer Stem Cells

Aside from their ability to induce pluripotency, the Yamanaka factors (OCT4, SOX2, KLF4, and c-MYC) [130], and other reprograming factors like NANOG or LIN28, have been widely implicated in tumorigenesis in various cancers including the prostate.

SOX2 is required for survival, pluripotency, clonogenicity, and self-renewal of ESCs. A relationship between SOX2 overexpression in tumorigenesis has been established in different types of cancer, including prostate [56] and its expression linked to tumor grade [58]. SOX2 is an epigenetic reprogramming factor and oncogene shown to regulate androgen-independent CRPC proliferation and evasion of apoptosis [57, 58] and to promote tumor metastasis by inducing EMT [59]. Further evidence suggests that SOX2 promotes self-renewal of the CSCs population by acting downstream of EGFR [131]. Importantly, in the recent years, SOX2 activity has been tightly associated to neuroendocrine transdifferentiation from prostate adenocarcinoma cells and the subsequent androgen independence of neuroendocrine prostate cancer phenotypes (NEPC). While the exact mechanisms remain unclear, substantial progress was made over the last couple of years. In particular, Russo and colleagues showed that SOX2 was expressed in NEPC murine models [60] whereas others found its expression restricted to NEPC areas of advanced human prostate cancer [61]. Recent studies by Bishop and collaborators at the Zoubeidi laboratory have shown that SOX2 is transcriptionally regulated by neural transcription factor BRN2 [132••], which in turn is negatively suppressed by the AR, hence revealing an AR-dependent suppression of cell differentiation toward a neuroendocrine AR-independent phenotype. Additional support to the central role of SOX2 in the emergence of NEPC and AR-independence after Enzalutamide treatment came from studies at the Ku and Mu and collaborators at the Sawyers and Goodrich laboratories [62••, 63••].

c-MYC (MYC) is a well-known oncogene proposed as a marker of disease progression in prostate cancer [133] and associated with prostate cancer recurrence and poor prognosis [134]. MYC activation cooperates with loss of PTEN to drive prostate cancer progression [135] and metastasis [136]. MYC proteins also drive epigenetic activation of gene expression in prostate cancer; the PRC2 member EZH2 is directly upregulated by MYC [137] and MYCN, which was shown to be a driver of NEPC [66] by inducing an EZH2-mediated transcriptional program [64]. Additionally, MYC expression was found to be regulated by the histone demethylase JMJD1A, controlling proliferation and survival of prostate cancer cells [138]. MYC also regulates the expression of histone demethylases PHF8 and KDMA3 in NEPC and CRPC [29]. Interestingly, while AR signaling in the normal prostate represses MYC expression, its expression is stimulated by AR during tumorigenesis, [139, 140]. It was also recently reported that MYC overexpression deregulates the AR transcriptional program by altering AR chromatin occupancy and H3K4me1 and H3K27me3 marks distribution, antagonizing clinically relevant AR target genes such as PSA [65].

## Oncogenic Pathways Involved in Epigenetic Regulations

Together with the AR, the oncogenic pathways most frequently altered in prostate cancer onset and progression are the RB, PI3K/AKT, and Ras/Raf pathways due to mutations in several members [72]. While the Ras/Raf pathway is activated in 43% of primary and 90% of metastatic prostate cancer, the triggering mechanisms remain to be fully understood. The Whitte laboratory demonstrated a synergistic interaction between Ras pathway activation and AR signaling that leads to elevated EZH2 expression and expand prostate cancer progenitor cells in vivo. It has been long suggested that this pathway is a major contributor of aggressiveness via the activation of EMT transcriptional programs. Nolan and colleagues proposed a model in which the secreted extracellular protein Hsp90 initiates ERK signaling and leads to the recruitment of EZH2 to the E-cadherin promoter and repression of E-cadherin expression, driving epithelial to mesenchymal transition (EMT) and invasion in prostate cancer cells [67]. Additionally, DAB2IP (disabled homolog 2 interacting protein) is a tumor suppressor Ras-GAP that negatively controls Ras-dependent mitogenic signals and modulates TNFα/NF-κB, WNT/β-catenin, PI3K/AKT, and androgen receptors pathways [68–70]. EZH2-induced DAB2IP silencing activates Ras and NF-kappaB and triggers metastasis [141, 142]. Data from our laboratory showed that concomitant activation of the PI3K and MAPk pathways in mice results in highly aggressive and fully metastatic tumors that are inherently castration resistant [143, 144]. Interestingly, by targeting the PI3K/MAPk pathways with small molecules in vivo, we demonstrated that the drug response network was highly enriched in epigenetic modulators, including SUV39H1, WHSC1, TOP2A, or UHRF1 [145], suggesting that epigenetic control of gene expression plays a central role in the aggressive phenotype imposed by the activation of Ras signaling. Accordingly, we have found that a core signature of chromatin modifiers and DNMTs drive the cancer cell intrinsic mechanisms of metastasis and CRPC (unpublished).

The retinoblastoma tumor suppressor gene RB1 is more commonly loss in metastatic and antiandrogen resistant prostate cancer (74% of cases) and NEPC (90% of cases) [71] than it is in primary tumors (34% of cases) [72]. It has been recently described an activity of Rb1 in the epigenetic regulation of expression, since RB1 directly repress the expression of Sox2 and Ezh2. Consequently, Rb1 loss in prostate cancer lead to EZH2 and Sox2 increase and gene expression widespread changes that leads toward a stem cell-like state that would facilitate the onset of metastasis, neuroendocrine transdifferentiation, and the acquisition of ADT resistance. The authors show that Ezh2 inhibition restores enzalutamide sensitivity in NEPC variants and recurrent prostate cancer cells by opposing lineage transformation [63••]. Furthermore, mutations in TP53 and RB1 tumor suppressor genes can promote a cellular plasticity state mediated by increased expression of SOX2 that, when it is compromised with antiandrogen therapy promotes resistance through lineage switching [62••]. It has also recently been shown that the Hedgehog (HH) signaling pathway and SOX2 co-operate in androgen-independent prostate cancer to promote carcinogenesis [146].

The PTEN/PI3K/AKT pathway is altered in 42% of primary and 100% of metastatic cases; loss of PTEN and activation of the PI3K/AKT signaling pathway are hallmarks of prostate cancer, and cooperate with the activation of the RAS/MAPK pathway to promote EMT and metastatic CRPC development. Epigenetically, it has also been shown that PTEN depletion contributes to a switch from a global H3K27 acetylatilation to H3K27 trimethylation, resulting in increased expression of EZH2 and decrease of the target genes DAB2PI together with negative regulator of cell growth p27^KIP1^ and p21^CIP1^ [147]. As mentioned above, increased AKT activity phosphorylates NSD2 at S172, preventing its degradation by CRL4^Cdt2^ E3 ligase, hence leading to its stabilization, which in turn upregulates RICTOR (mTORC2). This results in further enhancement of AKT signaling in a AKT/NSD2/mTORC2 positive feedback loop that sustains AKT signaling [1••].

Constitutive activation of TGF-β signaling is a well-recognized mechanism for induction of EMT and prostate cancer metastasis development. TGF-β1-induced EMT in prostate cancer is mediated by the histone methyltransferase RbBP5. RbBP5 is a conserved component of the COMPASS/-like complex, which catalyzes the trimethylation of histone H3 lysine 4 that is considered an epigenetic mark of actively transcribed genes. RbBP5 activity is in turn modulated by the binding of SMAD2/3, a downstream signaling factor to the TGF-beta pathway, to the Snail promoter [148]. Snail activates the EMT process by inhibiting transcription of E-cadherin via the recruitment to its promoter of the polycomb repressive complex 2 (PRC2) and the histone methylstranferase G9a, leading to repressive H3K27 and H3K9 methylation [149, 150]. An interesting new perspective was provided recently linking ERG signaling with TGF-β. Data suggest that ERG regulates the transcription of the transcription factor SOX4 and together they cooperate in TGF-β1-induced EMT of prostate cancer cells [151]. This is not surprising taking into account that the oncogenic role of SOX4 has been proposed in several other tumor types. In particular, SOX4 regulates EZH2 expression and chromating remodeling, and is a key component of the PI3K/AKT pathway in prostate cancer. In fact, SOX4 inhibition reduces AKT and β-catenin pathways activation and decreases prostate cancer invasiveness through positive feedback loop between SOX4 and PI3K-AKT-mTOR [152].

Finally, a tyrosine kinase, namely ACK1, has been found to link oncogenic signaling with epigenetic regulation. ACK1 was found upregulated in primary PCa and CRPC [72, 73], correlated with poor prognosis and reported to interact with AR to drive ADT resistance and CRPC growth [74]. A recent study demostrates that ACK1 regulates transcription of AR and AR-v7 via epigenetic regulation. In particular, ACK1 would phosphorylate histone H4 upstream of the AR transcription start site, recruiting the WRD5/MLL2 complex, therefore mediating H3K4 trymethylation and transcriptional activation. Inhibition of ACK1 with a small molecule inhibitor confirms that this epigenetic activity is required to maintain AR transcription and CRPC tumor growth [153].

## Drug Development on Epigenetic Regulators as Therapeutic Targets

Mounting evidence from basic and preclinical investigations suggest that targeting key components of the epigenetic machinery will have clinical benefit for cancer patients including prostate cancer ones. Yet, clinical development for those therapies is still very limited. On the one hand, this may be partly due to the inherent difficulty in targeting nuclear effector mechanisms. On the other hand, the fact that most epigenetic master regulators exert their functions over an extensive transcriptional network in a context-dependent manner makes it particularly challenging to achieve cancer cell specificity, thus resulting in significant toxicity. Despite these limitations, a number of drugs are currently in clinical trials at different phases, being BET bromodomain inhibitors, HMT/HDMT inhibitors, DNMT inhibitors, and HDAC inhibitors the focus of most intense drug development efforts. Table 2 summarizes the most relevant ongoing or recently completed clinical trials involving epigenetic drugs.

## Conclusion

In view of the accumulated evidenced supporting the key role of the dysregulated epigenome to prostate cancer onset and progression, three mechanisms emerge as the most significant contributors. First, a number of alterations in epigenetic master regulators result in enhanced transcriptional activity and pro-oncogenic role of the Androgen Receptor signaling. This is largely mediated by either remodeling of the chromatin to facilitate AR binding and assembly of the transcriptional complex and posttranslational modifications in the AR itself or essential co-factors resulting in gain of function features. Secondly, the aberrant activation of transcriptional programs tightly associated to developmental pathways and stem features, either via alterations in pioneering factors or pluripotency master regulators, contributes to the acquisition of treatment-resistant phenotypes that are highly aggressive. Finally, a significant number of alterations in epigenetic master regulators also result in the activation of oncogenic signaling pathways that contribute to the aggressiveness and androgen independence in advanced prostate tumors. In summary, the epigenome is emerging as an attractive and plausible target for anticancer therapy in general and prostate cancer in particular. While drug development is still limited, and faces inherent challenges associated with the unique nature of these targets, it seems evident that efficacy of such treatments will be maximized in combination with standard of care treatments for which most lethal prostate cancer ultimately develop resistant mechanism.
